# Screening for Polymorphism, Cyclodextrin Complexation, and Co-Crystallization of the Non-Steroidal Anti-Inflammatory Drug Fenbufen: Isolation and Characterization of a Co-Crystal and an Ionic Co-Crystal of the API with a Common Coformer

**DOI:** 10.3390/pharmaceutics17070842

**Published:** 2025-06-27

**Authors:** Hannah M. Frösler, Neo Refiloe Mancapa, Laura Catenacci, Milena Sorrenti, Maria Cristina Bonferoni, Mino R. Caira

**Affiliations:** 1 Centre for Supramolecular Chemistry Research, University of Cape Town, Rondebosch 7701, South Africa; hannah.frosler@alumni.uct.ac.za (H.M.F.); mncneo001@myuct.ac.za (N.R.M.); 2 Department of Drug Sciences, University of Pavia, 27100 Pavia, Italy; laura.catenacci@unipv.it (L.C.); milena.sorrenti@unipv.it (M.S.); mariacristina.bonferoni@unipv.it (M.C.B.)

**Keywords:** fenbufen, isonicotinamide, co-crystal, ionic co-crystal, cyclodextrins, thermal analysis, FT-IR spectroscopy, X-ray diffraction, solubility measurement

## Abstract

**Background/Objectives**: Increasing the solid-state landscape of an active pharmaceutical ingredient (API) by generating new crystalline forms (e.g., polymorphs, cyclodextrin (CD) inclusion complexes, co-crystals, and salts) can yield products with significantly enhanced biopharmaceutical properties (especially increased water solubility), thereby improving API delivery and extending its lifetime. The aim of this study was the isolation of new solid forms of the poorly water-soluble non-steroidal anti-inflammatory drug fenbufen (FBF), for which relatively few solid phases have been reported to date. Further motivation for the study is the recent finding that it has potential for repurposing to treat acute pancreatitis. **Methods**: Interventions for generating new solid forms of FBF included (a) polymorph screening with a variety of solvent media, (b) attempts to form solid inclusion complexes with the native cyclodextrins α-, β-, and γ-CD using various preparative methods, and (c) co-crystallization with a series of coformers to produce co-crystals and/or molecular salts. **Results**: No new polymorphic forms of FBF were identified, but screening with CDs resulted in isolation and characterization of a new solid inclusion complex with γ-CD. However, co-crystallization of FBF with the water-soluble coformer isonicotinamide yielded two new products, namely a 1:1 co-crystal and an unusual multi-component ionic co-crystal, whose aqueous solubility indicated significant enhancement of FBF solubility. **Conclusions**: Due to its extremely low water solubility, FBF presented challenges during the study aimed at modifying its crystalline form. However, two new supramolecular forms, a co-crystal and an ionic co-crystal, were isolated, the latter phase having potential for further formulation owing to its significantly enhanced solubility.

## 1. Introduction

Modification of the biopharmaceutical properties of an active pharmaceutical ingredient (API), especially that of low aqueous solubility which limits bioavailability, can be achieved via numerous crystal engineering approaches, including, e.g., the preparation of different polymorphic and solvated crystalline forms of the API [1], complexation of the API with cyclodextrins (macrocyclic oligosaccharides) [2,3,4], and co-crystallization of the API with biocompatible partner molecules (coformers) [5,6,7], which may result in a pharmaceutical co-crystal or a salt. The products of these interventions would generally display one or more favorable pharmaceutically relevant features compared to those of the untreated API, and could hence find potential application as new solid phases for drug formulation.

Screening for new polymorphs and solvates usually entails crystallization of the API from a series of solvents whose polarities span a wide range. This type of study is relevant from the point of view of drug preformulation, given the influence of crystal forms (polymorphism and solvatomorphism) on the different physical and chemical properties of biologically active compounds, including powder characteristics, melting point, enthalpy of fusion, dissolution behavior, and stability [8,9,10,11,12]. However, API solubility enhancement via this approach is typically only two-fold, or at maximum ~five-fold [13], and thus considerably more attention is given to the methods described below, which can achieve both significantly higher solubility enhancements and address other biopharmaceutical shortcomings of the API. The polymorphism and solvatomorphism of APIs are nevertheless important phenomena which in practice are encountered frequently.

Inclusion of API molecules within the hydrophobic cavities of cyclodextrin (CD) host molecules is a well-established strategy for increasing API solubility for improved drug delivery. Additional advantages of this technique include increased chemical stability of the API, reduction in gastrointestinal damage caused by acidic APIs, and masking of unpalatable API taste and odor [14,15,16,17]. For solid cyclodextrin inclusion complexes intended for medicinal formulation, comprehensive characterization is necessary, and structural elucidation by X-ray diffraction provides reliable proof of their authenticity.

Crystal engineering via co-crystallization of APIs with what are generally regarded as safe (GRAS) coformers to produce new multi-component co-crystals continues to be a vibrant field of research in both academia and in the pharmaceutical industry [18,19,20,21]. Both the API molecules and the coformer molecules in a co-crystal are electrically neutral. As indicated in the reviews cited above, the potential biopharmaceutical benefits that such co-crystal products may confer on APIs include, e.g., enhancement of their aqueous solubilities, leading to improved bioavailability, increased API stability and mechanical strength, apparent modification of the API melting-point via coformer variation, crystal morphology optimization to facilitate product manufacture, and elimination of API hygroscopicity. Multi-component co-crystals containing different API molecules (so-called ‘drug–drug co-crystals’) are also gaining increasing interest owing to their potential for targeting multiple disease states and possible synergistic effects that may ensue on their administration [22]. The reaction of an ionizable API and a coformer may result in salt formation rather than yielding a co-crystal. While pharmaceutical salts also generally display improved solubility relative to that of the API, the distinction at the molecular level between ionic co-crystals/salts and co-crystals is critical for the registration of drug products, and hence reliable analysis via spectroscopic and X-ray diffraction methods are required to distinguish them unequivocally.

**Figure 1:**
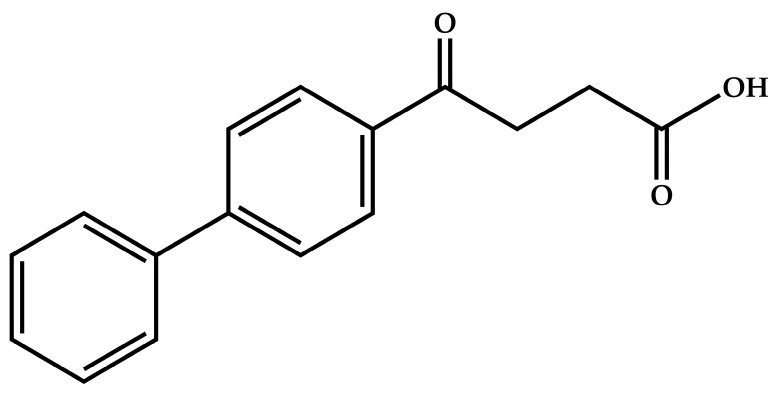
The chemical structure of fenbufen.

The present study focuses on our attempts to use the approaches described above with a view to addressing the extremely low aqueous solubility of the potent non-steroidal anti-inflammatory drug (NSAID) fenbufen, whose structure is shown in Figure 1. Fenbufen (IUPAC name 4-oxo-4-(4-phenylphenyl)butanoic acid) is a Class II drug in the Biopharmaceutical Classification System, having low aqueous solubility, namely 0.0022 mg/mL at 25 °C [23], but high permeability.

Fenbufen (hereinafter FBF) is a well-established NSAID. It acts as a prodrug whose major metabolite, 4-biphenylacetic acid, is a potent, non-selective inhibitor of the enzyme cyclooxygenase (COX-1 and COX-2), thereby preventing the synthesis of prostaglandins which cause inflammation [24,25]. The analgesic and antipyretic effects of FBF are used to treat patients with osteoarthritis, rheumatoid arthritis, tendinitis, ankylosing spondylitis, and post-operative pain [25]. Despite certain adverse side effects of FBF (including hepatotoxicity, which is common for NSAIDs [26]), there is ongoing interest in this API for both its current applications and possible future applications. The X-ray crystal structure of FBF has been reported in the Cambridge Structural Database (CSD [27]) with refcode SAFNIW. The asymmetric unit is a pseudo-centrosymmetric carboxylic acid dimer. With regard to its co-crystallization with coformers, the X-ray structures of four co-crystals with amine derivatives have been reported in the CSD with refcodes UCEROM, UCERUS, UCESAZ, and UCETOO. However, as the respective coformers are not APIs, the relevance of these co-crystals in the context of the present study is not significant. The same applies to the occurrence of deprotonated FBF as a ligand in several metal compounds [27]. However, a recent pertinent report in the context of medicinal application is that of the synthesis and characterization of a drug–drug molecular salt containing the FBF anion and the cation of the antimalarial drug pyrimethamine [28]. The authors of this study reported enhanced solubility of the antimalarial in the form of the salt. Numerous APIs, including NSAIDs, have been included in CD molecules and the resulting complexes have been characterized by physicochemical methods. Earlier solution- and solid-state studies (~1990–2015) of the inclusion of FBF in β- and γ-cyclodextrins using NMR spectroscopy, FT-IR spectroscopy, and molecular modeling were reported. However, the recent systematic review on CD inclusion of NSAIDS covering the period 2010–2020 lists 24 different NSAIDs [29], but FBF is absent from the list. Several NSAIDs, including FBF, were recently used as model guests for analysis of their complexation with β-CD in solution using induced circular dichroism, NMR spectroscopy, and molecular modeling [30]. However, there is a lack of structural information for solid CD inclusion complexes of FBF. Finally, it was recently reported that NSAIDs, including FBF, exhibit a novel anti-inflammatory mechanism, namely the inhibition of multi-functional caspases (intracellular proteases), resulting in (inter alia) reduced cell death [31]. Since caspases are newer targets for inhibition, NSAID repurposing could therefore become more widespread. In a more recent report, FBF was accordingly shown to inhibit specific caspases, thereby reducing the inflammatory response in mice with severe acute pancreatitis (SAP), a disease with a high morbidity rate [32].

An assessment of the results of the present studies follows. Our screening for polymorphism of FBF yielded no new crystal forms. Furthermore, using a variety of methods for complexing FBF with α-, β-, and γ-cyclodextrins to yield solid inclusion compounds, the only solid complex isolated was that with γ-cyclodextrin obtained via the kneading method, for which structural information was deduced from powder X-ray diffraction (PXRD). However, experiments aimed at co-crystallization of FBF with the water-soluble coformer isonicotinamide via liquid-assisted grinding (LAG) and co-precipitation methods were successful, yielding two new products. The first was an FBF-isonicotinamide co-crystal with 1:1 stoichiometry, which was analyzed by thermal and single-crystal X-ray diffraction (SCXRD) methods. The second product was an unusual multi-component crystal whose asymmetric unit comprises one FBF molecule, one FBF anion, one protonated isonicotinamide molecule, two neutral molecules of isonicotinamide, and a water molecule. Comprehensive characterization of the latter crystalline phase was undertaken using thermal analysis, powder and single-crystal X-ray diffraction methods, FT-IR spectroscopy, and solubility determination in both water and a simulated intestinal fluid. Significant solubility enhancement of FBF in the form of the multi-component ionic co-crystal was achieved in aqueous medium.

## 2. Materials and Methods

### 2.1. Materials

Fenbufen (FBF, mol.wt. 254.28 g mol^−1^, purity 96%) was purchased from Sigma-Aldrich (St. Louis, MO, USA) and Sigma-Aldrich (Johannesburg, South Africa). All coformers used in co-crystallization experiments were purchased from Sigma-Aldrich Chemie GmbH (Steinheim, Germany). The following cyclodextrins were purchased from CycloLab (Budapest, Hungary): α-cyclodextrin (α-CD, mol.wt. 972.9 g mol^−1^, purity 97%), β-cyclodextrin (β-CD, mol.wt. 1135.0 g mol^−1^, purity 98%), and γ-cyclodextrin (γ-CD, mol.wt. 1297.2 g mol^−1^, purity 97%). All other materials and solvents were of analytical-reagent grade.

### 2.2. Methods

#### 2.2.1. Polymorph Screening

The commercial product was first sieved and the granulometric fraction with particle size < 250 µm was collected for further experiments. Screening for polymorphism involved the dissolution of FBF in several selected solvents. In each case, approximately 50 mg of FBF was dissolved with magnetic stirring and heating to a temperature just below that of the boiling point of the solvent. The clear solutions were filtered and left to crystallize by spontaneous cooling to room temperature. The crystalline solids were filtered, dried in a dryer containing P_2_O_5_ as desiccant, and characterized by recording their onset and peak temperatures of melting as well as their fusion enthalpies by differential scanning calorimetry (DSC) using a Mettler STAR^e^ system (Version 16.20c) (Mettler Toledo, Milan, Italy) equipped with a DSC821^e^ Module and an Intracooler device for sub-ambient temperature analysis (Julabo FT 900, Seelbach, Germany). Further characterization included Fourier transform infrared (FT-IR) mid-IR spectroscopy (650–4000 cm^−1^ range, 64 scans, resolution 4 cm^−1^) using a Spectrum One spectrophotometer (Perkin Elmer, Monza, Italy). The instrument employed a MIRacle^TM^ attenuated total reflectance (ATR) device (Pike Technologies, Madison, WI, USA). The solid samples (about 4 mg of powder for each sample to obtain quantitatively comparable data) were pressed on an ATR crystal of ZnSe for spectral acquisition in transmittance mode.

#### 2.2.2. Complexation with CDs

Evaluation of the feasibility of complexation between FBF and the native cyclodextrins (CDs), namely α-, β-, and γ-CD, was undertaken using a variety of preparative methods, viz., kneading (KN), co-precipitation (CP), and co-evaporation using a rotary evaporator (Buchi Italia, Cornaredo, Italy) under reduced pressure (RV). Physical mixtures (PMs) were prepared by homogeneously mixing equimolar amounts of FBF with each native CD, previously sieved to collect the particle size fraction < 250 µm, in a turbula mixer for 20 min. The solids isolated from these experiments were assessed using DSC and FT-IR spectroscopic analyses using the same instrumentation as listed above for polymorph screening. Attempts at isolating single crystals of FBF-CD complexes involved the preparation of saturated solutions of the CD at 70 °C in MilliQ^®^ (Merck Millipore, Cape Town, South Africa) water to which equimolar quantities of FBF were added in small increments while stirring the solutions for a minimum of 4 h or overnight. The solutions were subsequently filtered (0.45 µm nylon microfilter) and allowed to evaporate slowly.

#### 2.2.3. Co-Crystal Screening

Prior to further experimental tests for FBF–coformer co-crystal formation, virtual screening using the molecular complementarity screening tool in the Cambridge Structural Database (CSD) Materials module of the program Mercury [33] was conducted to identify coformers that would have potential for co-crystal formation with the FBF molecule. Following the upload of the structure of FBF, this API was screened against a list of ‘generally recognized as safe’ (GRAS) coformers. The screening tool yielded ‘pass’ or ‘fail’ indicators depending on whether co-crystal formation is likely or not with each FBF–coformer combination. Priority was given to coformers with a ‘pass’ indicator and their number was limited by their availability in the laboratory. For experimental screening, liquid-assisted grinding (LAG) of each equimolar FBF–coformer mixture was employed. Generally, the LAG process was performed for 20 min with each of the solvents (ethanol, ethyl acetate, and acetonitrile) independently, with 15 µL additions at the rate of approximately three increments per minute to 10 mg FBF and an equimolar amount of coformer. Manual grinding was employed using an agate mortar and pestle, and a calibrated microsyringe was used for the solvent additions. CP experiments via both slow evaporation and slow cooling procedures were conducted to grow single crystals of co-crystallized products for full X-ray structure determination. Generally, equimolar masses of the API and the coformer were weighed accurately and then dissolved in separate vials in the chosen common solvent by placing the vials on a hot plate using magnetic stirring, the solution temperatures being maintained at ~10 °C below the boiling point of the solvent. After addition of the coformer solution to that of the API, the resulting solution was stirred for 4 h (or overnight if necessary) and filtered (0.45 µm nylon microfilter).

#### 2.2.4. Co-Crystal/Ionic Co-Crystal Analysis

##### Powder X-Ray Diffraction (PXRD)

Each product from LAG was analyzed using powder X-ray diffraction (PXRD) and its pattern was compared with those of the individual starting materials to ascertain whether a new crystalline phase had been produced. A Bruker D8 Advance X-ray diffractometer and a Bruker D2 Phaser desktop powder diffractometer (Billerica, MA, USA) were used to record PXRD patterns with CuKα_1_ radiation (λ = 1.5406 Å). X-ray generator settings were 30 kV and 10 mA. Crystalline samples were finely ground to minimize preferred orientation, placed on a zero-background sample holder and scanned from 4.0° to 40.0° 2θ with a step size of 0.0164°.

##### Single-Crystal X-Ray Diffraction (SCXRD)

Reflection intensities were measured on Bruker APEX II CCD Duo and Bruker D8 Venture diffractometers (Madison, WI, USA) using MoKα radiation (λ = 0.71073 Å). Crystals were mounted on nylon cryoloops and cooled in a constant stream of nitrogen vapor using an Oxford Cryostream-800 (Oxford Cryosystems, Long Hanborough, UK). The structures were solved by direct methods and refined by full-matrix least-squares on F^2^. All non-hydrogen atoms were treated anisotropically. Particular attention was given to the unequivocal location of hydrogen atoms, given the possibilities of co-crystal or salt formation. All H atoms were therefore located in difference Fourier maps and were included in the refinements in a riding model with isotropic thermal displacement parameters in the range of 1.2–1.5 times those of their parent atoms. Further details of the data-reduction, structure solutions, refinements, and relevant software used are listed in the Crystallographic Information Files available from the Cambridge Crystallographic Data Centre (see details in the Supplementary Materials list).

##### Hot Stage Microscopy (HSM)

HSM was performed by heating crystalline samples on a Linkam THM600 (Linkam Scientific Instruments, Salfords, UK) hot stage in the temperature range of ~20 to 350 °C using a Linkam TP92 controller unit set at 10 K.min^−1^. A Sony Digital Hyper HAD (Sony, Johannesburg, South Africa) video camera was used to capture significant events during sample heating and the data were processed using the imaging program AnalySIS [34].

##### Thermogravimetric Analysis (TGA)

TGA was performed using a TA-Q500 instrument (New Castle, DE, USA). Accurately weighed samples with masses in the range of 3 to 5 mg were placed in an alumina crucible and were heated at 10 K.min^−1^ while a constant stream of dry nitrogen purge gas flowed through the instrument at 60 mL.min^−1^. TGA curves were recorded from 20 to 350 °C and analyzed using TA Universal analysis 2000 software [35].

##### Differential Scanning Calorimetry (DSC)

For DSC measurements a TA Discovery DSC 25 instrument (New Castle, DE, USA) was used. Accurately weighed samples in the range of 1–2 mg were placed in aluminium pans and heated at 10 K.min^−1^. For data analysis, TRIOS software (Version 4.1.0.31739) was employed [36].

##### Fourier-Transform Infrared (FT-IR) Spectroscopy

Spectra were collected on a PerkinElmer Spectrum Two instrument (Waltham, MA, USA) fitted with an attenuated total reflectance (ATR) attachment (Waltham, MA, USA). After the diamond crystal was cleaned, the background radiation spectrum was recorded. The sample material was then placed on the diamond and held in tight contact after sealing. Spectra were recorded over the range of 4000–400 cm^−1^ with a resolution of 4 cm^−1^ and 32 accumulations.

##### Elemental Analysis (EA)

The percentages of C, N, and H in the samples were recorded on an Elementar Vario EL Cube Elemental Analyzer (Elementar Analysensysteme GmbH, Langenselbold, Germany). Accurately weighed samples with masses in the range of 4–12 mg were analyzed. Reliability of the results was based on the accuracy of the elemental percentages of standards which were analyzed between sample measurements.

##### Solubility Studies

The material of interest (viz., the multi-component solid containing the API) was added to an accurately measured volume of the medium (1.00 mL of pure water or fasted state simulated intestinal fluid (FaSSIF) solution buffered at pH 6.5) in accurately weighed incremental amounts of approximately 0.10 mg each to eventually achieve saturation. Following the addition of the first incremental amount and its complete dissolution with stirring (at 250 rpm) at a constant temperature (25 ± 1 °C), further incremental masses were added and dissolved until it was evident that further addition led to precipitation. The stirred solution was monitored for 72 h. The total mass of the sample added was estimated by calculating the average of (a) the accumulated mass added, including the penultimate addition (which dissolved) and (b) the former mass plus the final incremental mass (that resulted in precipitation). The mass of the API present in the sample was calculated from its mass fraction in the sample multiplied by the sample mass. The solubility enhancement of the API was then calculated as the ratio of the concentration of the API in the product and the solubility of the API. The solubility experiments were performed in duplicate.

## 3. Results and Discussion

### 3.1. Polymorph Screening

**Figure 2:**
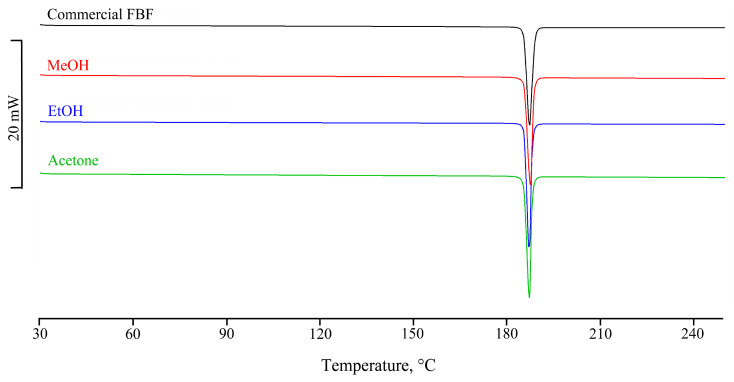
Consistent appearance of sample DSC curves for the recrystallized solids.

The DSC curve and the FT-IR spectrum of the sieved commercial sample of FBF were recorded to serve as references in the identification of any new polymorphs. The DSC curve (Figure 2) was recorded in the temperature range between 30 and 350 °C and showed a single endothermic effect at 185.5 ± 0.4 °C (T_onset_ = 185.1 ± 0.6 °C; ΔH_m_ = 178 ± 1 J g^−1^) associated with the fusion of the sample. The subsequent endothermic effect at 280 °C is attributable to the melt decomposition, confirmed by a TGA mass loss of about 78% recorded from 195 °C. Recrystallization solvents included methanol (MeOH), ethanol (EtOH), acetone, dimethyl sulfoxide, isopropanol, acetonitrile, ethyl acetate, diethyl ether, and chloroform, as well as solvent mixtures MeOH/water and EtOH/water in three different volume ratios. Table S1 (Supplementary Materials) lists these 15 solvent media, their volumes in mL/50 mg, and the percentage yields of the solid products. All of the recrystallized samples displayed the same thermal behaviour (fusion onset and peak temperatures, and fusion enthalpies) as that recorded for the commercial FBF sample (Table S2, Supplementary Materials), thereby indicating that no new polymorphic forms were evident. Figure 2 shows the consistent appearance of sample DSC curves.

**Figure 3:**
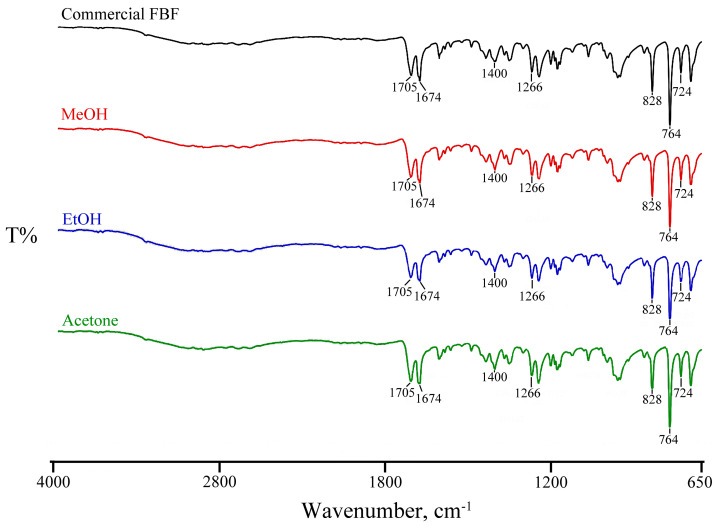
Consistent appearance of sample FT-IR spectra for recrystallized solids.

The FT-IR analysis of FBF (Figure 3) was recorded and the following prominent assigned bands included those due to C-H and O-H stretching vibrations in the 3500–2500 cm^−1^ range, the C=O stretching vibrations of the carboxylic acid function at 1705 cm^−1^ and that of the ketonic carbonyl group at 1674 cm^−1^, bands attributable to the methylene bending vibrations between 1470 and 1430 cm^−1^, and out-of-plane bending vibrations of the C=C-H aromatic rings at 828, 764, and 724 cm^−1^. As is evident from Figure 3, the consistent appearance of the sample FT-IR spectra confirms that no new polymorphs of FBF were identified under the conditions described above.

### 3.2. Attempted Complexation of FBF by Native Cyclodextrins

**Figure 4:**
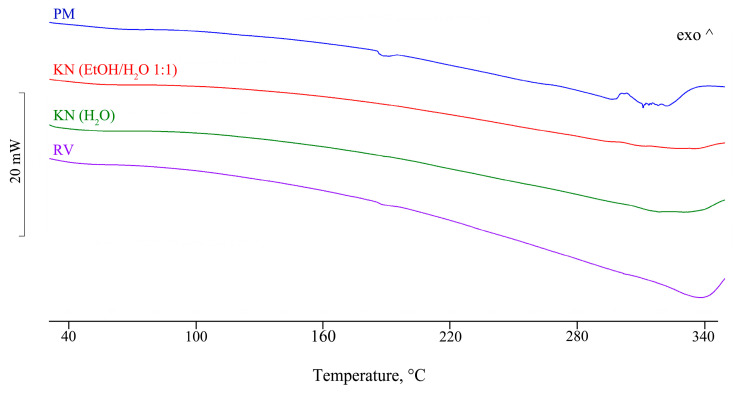
DSC profiles for samples of binary products γ-CD—FBF prepared by various methods.

One of the goals of this study was the isolation of single crystals of CD-FBF complexes and structure determination by SCXRD, as no CD-FBF crystal structures have been deposited in the CSD [27]. Details of the methods used in attempts to complex FBF with α-CD, β-CD, and γ-CD, namely kneading (KN), co-precipitation (CP), and co-evaporation using a rotary evaporator under reduced pressure (RV), are provided (Table S3, Supplementary Materials). Using these methods, experiments with the host compounds α-CD and β-CD indicated no affinity for their complexation with FBF. However, positive results were obtained with γ-CD, as reported below. To test the possible interaction between γ-CD and FBF, four samples were prepared for DSC analysis. Figure 4 shows the DSC curves for the physical mixture (PM), those for the products obtained by kneading (KN) under different conditions, and that for the sample obtained by co-evaporation using a rotary evaporator under reduced pressure (RV).

**Figure 5:**
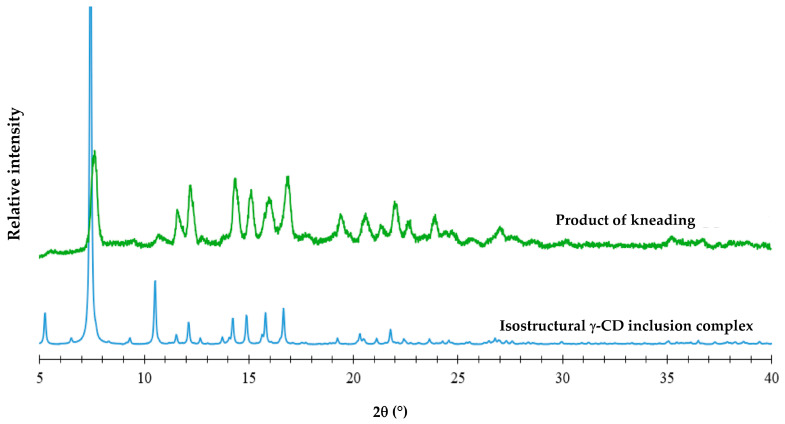
The PXRD pattern of the product of kneading an equimolar mixture of γ-CD and FBF (green) and the calculated PXRD pattern of an isostructural γ-CD complex (blue).

For the PM, the endotherm at 187 °C corresponds to the melting of FBF, the enthalpy value being reduced by 35% relative to that of pure FBF. However, this endotherm is not present for either of the KN samples, thus providing evidence of inclusion of the FBF molecule in the cavity of the host γ-CD molecule. For the RV sample, the reduction in the FBF melting enthalpy value was 84%. The interpretation of the thermal data was confirmed by corresponding FT-IR spectra for the samples, which showed the disappearance of some spectral bands and significant frequency shifts (Figure S1 and Table S4, Supplementary Materials). For all of the samples prepared by the CP method, the solid phase isolated was FBF only; this is attributed to the extremely low aqueous solubility of FBF and the absence of the CD component, which remained in the solution after the filtration step. The objective of using SCXRD analysis to determine the detailed solid-state structural features of CD inclusion complexes of FBF was not fulfilled due to the lack of single crystals, despite attempts to grow them. However, in an effort to obtain structural information for the γ-CD-FBF complex mentioned above using PXRD, an equimolar mixture of the two reactants underwent KN for 20 min using a total of 80 µL MilliQ^®^ waterand the PXRD pattern of the product was recorded (Figure 5).

From the close match of the PXRD profiles above, it can be deduced that the product of KN is isostructural with that of a known γ-CD inclusion complex (namely the γ-CD bentazon clathrate tetrahydrate, with Cambridge Structural Database (CSD [27]) refcode KECWOI). Thus, based on the known data for KECWOI, it can be concluded that the new γ-CD·FBF complex crystallizes in the tetragonal system, space group *P*42_1_2 [37], which is the most common space group for γ-CD complexes. The close resemblance between the PXRD patterns also suggests that the γ-CD·FBF complex has 1:1 stoichiometry, corresponding to the equimolar mixture of reactants employed in the preparation. However, for confirmation, the DSC curves of the KN products of PMs with starting reactants γ-CD and FBF in the molar ratios 1:2, 1:1, and 2:1 were recorded. Given that the melting point of FBF is 186.7 °C, any excess of FBF should have appeared as an endothermic peak in the DSC curve. Examination of the DSC curves (Figure S2, Supplementary Materials) showed that the 1:2 composition displayed a small endotherm at 186.5 °C, indicating pure FBF excess, while neither of the 1:1 nor the 2:1 compositions showed any trace of fusion, these results being consistent with the 1:1 host–guest stoichiometry of the complex. Representative TGA and DSC curves for the fully hydrated complex were also recorded (Figure S3, Supplementary Materials). A 14.2 ± 0.5% TGA mass loss between 20.8 and 175 °C due to complete dehydration of the complex was recorded, corresponding to 14.3 ± 0.6 water molecules per GCD molecule. Based on the cumulative quantitative results, the formula for the new complex is thus γ-CD·FBF·14.3 H_2_O, the full chemical formula being C_48_H_80_O_40_∙C_16_H_14_O_3_∙14.3 H_2_O.

### 3.3. Co-Crystal and Ionic Co-Crystal Forms Containing FBF

#### 3.3.1. Co-Crystal FBF·ISONIC: Synthesis and Analysis

**Figure 6:**
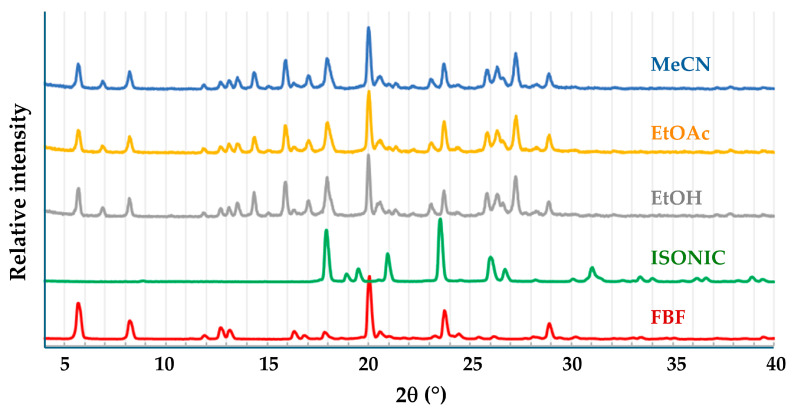
PXRD patterns of the starting materials FBF and ISONIC, and those of the products of LAG with the solvents indicated.

The presence of a carboxylic acid group in FBF indicated that suitable coformers for possible co-crystal formation would be biocompatible carboxylic acids and amides, since known co-crystals are frequently based on acid-acid and acid-amide supramolecular synthons [5]. Initial experiments involved the use of the well-known water-soluble coformer isonicotinamide (IUPAC name pyridine-4-carboxamide). Figure 6 shows the PXRD patterns of the products of liquid-assisted grinding (LAG) of equimolar amounts of FBF (10.0 mg) and isonicotinamide (ISONIC, 4.8 mg) using three solvents, namely ethanol, ethyl acetate, and acetonitrile.

The patterns of the products are identical, but on careful inspection, they appear not to be simply the sum of the patterns of the starting materials due to the appearance of several new significant peaks and the disappearance of several peaks of ISONIC. To obtain single crystals of the product, a co-precipitation experiment using EtOAc as solvent, and the same amounts of FBF and ISONIC as those used for LAG, yielded colorless crystals. HSM indicated a melting point onset temperature of 139 °C, complete fusion at 150 °C, and no indication of solvation. TGA confirmed the lack of solvation, indicating mass loss commencing only after the melting event. Using DSC, one endothermic peak was observed with a temperature range of 129–155 °C, peaking sharply at 143 °C, and the fusion enthalpy was 118.8 J g^−1^. The peak temperature of 143 °C differed significantly from the melting temperatures of FBF (186–187 °C) and ISONIC (155–157 °C), thus indicating a new crystalline phase (Figures S4 and S5 in the Supplementary Materials show relevant thermal results).

**Figure 7:**
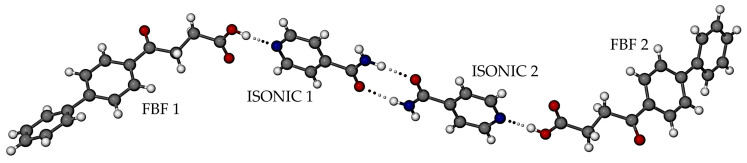
The asymmetric unit in the FBF·ISONIC co-crystal.

The structure of the new phase was determined by SCXRD, which revealed a new co-crystal with 1:1 FBF:ISONIC stoichiometry (Figure 7), crystallizing in the triclinic system, space group *P*1 (Table S5, Supplementary Materials lists crystal data, data-collection details, and refinement parameters). The asymmetric unit (ASU) comprises four crystallographically indpendent molecules, namely two distinct FBF molecules and two distinct ISONIC molecules. Dimerisation of the ISONIC molecules is maintained by the two intermolecular amide-amide N-H···O hydrogen bonds, the resulting homosynthon having the graph set description $R_{2}^{2}\left(8\right)$ and the motif in Figure 7 is completed by the attachment of the terminal FBF molecules to the pyridine nitrogen atoms via the respective -COOH···N hydrogen bonds. The motif adopts a pseudo-centrosymmetric conformation.

**Figure 8:**
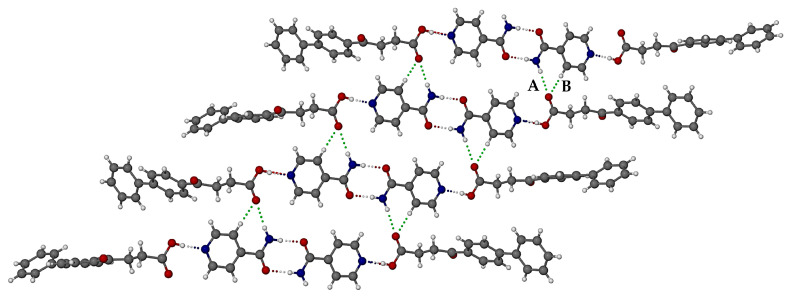
Mode of assembly of the motifs in the FBF·ISONIC co-crystal.

A portion of the extended crystal structure (Figure 8) shows that the four ISONIC dimers in these motif units are coplanar. The resulting layer is assembled via hydrogen bonding between the motifs, namely amide(N-H)···O=C(carboxylic) H-bonds and prominent pyridyl(C-H)··· O=C(carboxylic) H-bonds (respective bond labels A and B in Figure 8). The three-dimensional structure is assembled via close stacking of layers of the type shown in Figure 8.

**Figure 9:**
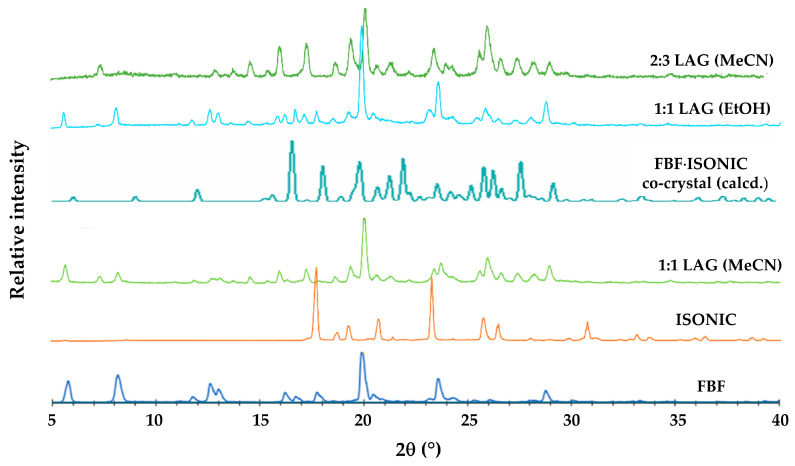
PXRD patterns used to glean information concerning the new FEN-ISN phase.

The calculated PXRD pattern for the FBF·ISONIC co-crystal is shown in Figure 9 and it is distinctly different from the common PXRD pattern of the products shown in Figure 6, indicating that the single crystals obtained by the co-precipitation method represent a different crystalline phase. Furthermore, this outcome suggests that the LAG process resulted in only a small extent of co-crystallization of the starting materials and that the additional peaks observed in the LAG product signified yet another phase emerging.

#### 3.3.2. Ionic Co-Crystal Form: Synthesis and Analysis

Following the characterization of the FBF·ISONIC co-crystal, the intention was the determination of its aqueous solubility to establish possible enhancement in the solubility of its FBF content. However, an unexpected situation arose, whereby all further attempts to produce a new batch of the co-crystal phase for solubility measurement were unsuccessful. Numerous additional experiments with various solvents and different reaction conditions, including recrystallization of LAG products, followed over a period of one year, but the desired co-crystal phase remained elusive (this outcome is revisited in a later section). However, given the fact that we isolated at least one new pharmaceutically relevant co-crystal of this API, it was obvious that testing additional GRAS co-formers for co-crystallization with FBF could be a fruitful exercise. To this end, computational co-crystal screening with FBF as the API using the Molecular Complementarity Screening tool module in Mercury [33] was employed. This screening procedure indicated 23 coformers as having potential for co-crystallizing with FBF. A small subset of these_,_ together with other carboxylic acid and amide coformers available in the laboratory, including isonicotinamide (a total of 19 coformers) were finally selected (Table S6, Supplementary Materials) for both LAG and co-precipitation experiments. For brevity, the outcomes of these experiments were as follows: all 19 co-precipitation experiments yielded only FBF as the solid product; LAG experiments yielded PMs in all cases, except one, which involved (once again) isonicotinamide as coformer. Figure 9 summarises the phases involved and the final identification of the new product, namely an unusually complicated, multi-component ionic co-crystal.

Careful analysis of the PXRD patterns above indicated that the 1:1 LAG experiments produced small peaks possibly representing excess FBF, suggesting a new phase with FBF:ISONIC molar ratio 1:>1. Thereafter, a single crystal was isolated from a co-precipitation experiment using MeCN (1:1 molar ratio). The crystal intensity data were collected at 100(2) K and the monoclinic system was indicated by the 2/m Laue symmetry of the reflection intensities. The space group was determined as *P*2_1_/c, based on the systematic absences h0l: l = 2n + 1; 0k0: k = 2n + 1 (Table S7, Supplementary Materials lists crystal data, data collection details, and refinement parameters). Structure solution of this crystal indicated a 2:3 FBF:ISONIC molar ratio for the crystal, and thus all subsequent LAG and co-precipitation reactions intended to produce this new phase in bulk were performed using this stoichiometric ratio. As indicated earlier, the 2:3 LAG experiment yielded a product with a unique PXRD pattern not observed previously. The composition of the ASU and a well-defined reproducible preparative method for isolating crystals of this new phase appear in Figure S6, Supplementary Materials.

**Figure 10:**
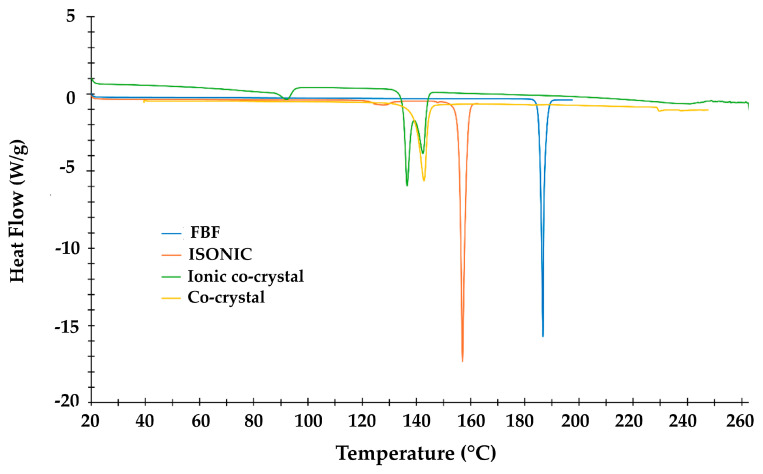
Overlaid DSC curves for four crystalline species.

HSM analysis of the new crystals recorded at 2 K.min^−1^ displayed multiple events, including bubble release at ~90 °C, later correlated with the mass loss in TGA of 1.72 ± 0.02% corresponding to 0.85 ± 0.01 water molecules per ASU of the ionic co-crystal. Figures S7 and S8 show HSM and TGA analytical results (Supplementary Materials). The DSC results are shown in Figure 10, and they include curves for the four species indicated, whose onset temperatures (T_on_), peak temperatures (T_peak_), and enthalpy values are listed in Table 1. For the new ionic co-crystal, the first endotherm (T_on_ = 86.9 °C) signifies the dehydration step (confirmed by both HSM and TGA). Two overlapping peaks (T_on_ = 135.3 °C and 139.0 °C) are attributed to fusion of the anhydrous compound with simultaneous emergence of a new crystal phase, the second endotherm (T_peak_ = 142.3 °C) occurring at nearly the same temperature as that for fusion of the 1:1 FBF·ISONIC co-crystal (T_peak_ = 143.3 °C). However, this is coincidental, as the new ionic co-crystal phase has still not incurred further mass loss at this temperature, and the 2:3 FBF:ISONIC composition should therefore still be intact. We also note the order of increasing thermal stabilities, namely anhydrous ionic co-crystal ≈ co-crystal < ISONIC < FBF. The significantly lower melting point of the ionic co-crystal was noted and considered as favorable for potentially enhancing the solubility of the API.

**Figure 11:**
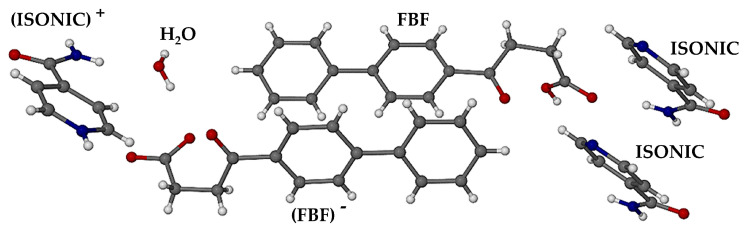
The asymmetric unit in the ionic co-crystal form.

The asymmetric unit (ASU) of the ionic co-crystal phase, established by SCXRD, is shown in Figure 11. It consists of six components, namely one FBF molecule, one FBF anion, two molecules of isonicotinamide, one protonated isonicotinamide molecule, and a water molecule.

All hydrogen atoms in the ASU were located unequivocally, confirming the composition of the crystal. Deprotonation of one of the FBF molecules was confirmed by the lengths of the C-O bonds of the resulting carboxylate group, namely 1.243(3) and 1.266(3) Å. Instead, for the neutral FBF molecule, the carboxylic acid bond lengths C=O and C-O are 1.215(2) and 1.322(2) Å, respectively, and protonation of the pyridyl nitrogen atom of only one coformer was observed. The estimated TGA mass loss for dehydration of the crystal indicated a water content of ~0.85 of a water molecule in the ASU. However, following the refinement of the water molecule with this site occupancy, assignment of unit occupancy resulted in very satisfactory refined anisotropic thermal displacement factors for the water ocygen atom, confirming reasonable assignment of the crystal as a monohydrate of a complex ionic co-crystal, hence having a final abbreviated formula (FBF) (FBF)^−^ 2(ISONIC) (ISONIC)^+^ H_2_O, and a chemical formula (C_16_H_14_O_3_)(C_16_H_13_O_3_)^−^ 2(C_6_H_6_N_2_O)(C_6_H_7_N_2_O)^+^∙H_2_O. Elemental analysis of single crystals yielded the values %C 66.9, %H 5.52, and %N 9.35, which are all within 0.4% of the calculated values (%C 67.25, %H 5.42, and %N 9.41).

**Figure 12:**
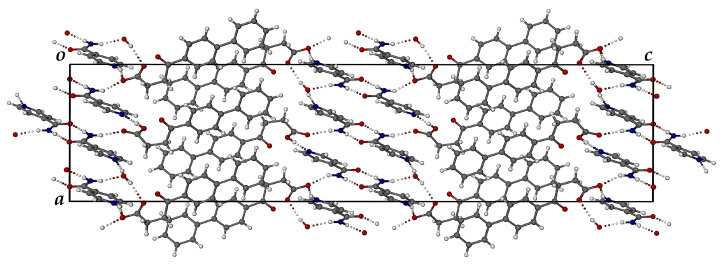
The [010] projection of the crystal packing in the ionic co-crystal form.

Figure 12 shows the crystal packing diagram viewed along the [010] direction. Two distinct domains are evident, namely one comprising FBF molecules and anions, and the other consisting of protonated ISONIC units and neutral ISONIC molecules. In the latter domain in the central part of the unit cell, there are alternating layers of H-bonded ISONIC dimers and H-bonded ISONIC-ISONIC^+^ units. The H-bonded ISONIC dimers are formed via amide-amide homosynthons with graph set descriptor $R_{2}^{2}\left(8\right)$ involving N-H···O hydrogen bonds, and the dimers are also H-bonded to water molecules as well as the carbonyl oxygen atoms of FBF molecules. The H-bonded ISONIC-ISONIC^+^ units display analogous features. Each water molecule is tightly bound in the crystal by three intermolecular H-bonds.

In addition to the copious hydrogen bonding interactions shown above, including occurrences of charge-assisted H-bonding due to the proton transfer, there are numerous intermolecular C-H···π and π-π interactions that stabilize the crystal structure. The FT-IR spectrum of the ionic co-crystal form shows features that clearly differ from those of FBF and ISONIC (Figure S9, Supplementary Materials).

**Figure 13:**
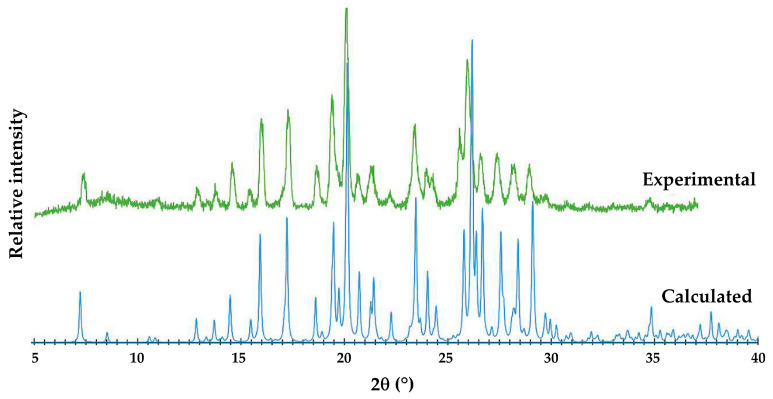
Experimental and calculated PXRD patterns of the ionic co-crystal form.

Figure 13 shows that the single crystal selected for SCXRD (calculated PXRD) is representative of the bulk material (experimental PXRD). Small differences in corresponding peak angular positions, especially at high 2θ angles, are due to the differences in temperature, namely 100 K and 294 K, respectively.

A plausible reason for the inability to recover the (anhydrous) 1:1 co-crystal phase is the possible presence of very small quantities of atmospherically absorbed water in recrystallization solvents subsequently used in co-precipitation experiments intended to regrow the co-crystal. This could encourage crystallization of the ionic co-crystal, which features both tightly bound water molecules as well as stronger, charge-assisted hydrogen bonding. A major goal of the study was to establish whether the incorporation of FBF in the new ionic co-crystal form would enhance the solubility of the API. Due to the extremely low aqueous solubility of FBF, difficulties arose with attempts to measure its concentration accurately using UV-Vis spectroscopy and high-performance liquid chromatography (HPLC), and therefore, the gravimetric technique described in “Section Solubility Studies” was used. Table 2 shows the results obtained for the solubility of FBF at 25 °C in the form of the ionic co-crystal in both pure water and fasted state simulated intestinal fluid (FaSSIF) following sample dissolutions in 1.00 mL of each medium. The value of 0.68 mg in Table 2 is thus the amount of FBF in the ionic co-crystal, obtained by multiplying the mass fraction of FBF (0.57) by the mass of the dissolved sample (1.19 mg).

The solubility enhancement factor for FBF in water as medium is thus ~300-fold. Futhermore, the solubility of pure FBF in FaSSIF is ~10 times that of FBF in the form of the ionic co-crystal in water.

## 4. Conclusions

The meager solid-state landscape of the non-steroidal anti-inflammatory drug fenbufen (FBF) is reflected in entries in the Cambridge Structural Database [27] that include only crystal structures of one FBF crystal form and four co-crystals whose coformers are not medicinally relevant. In addition, the deprotonated FBF species features as a ligand in four metal complexes that are likewise not intended for pharmaceutical applications. Thus, the lack of alternative crystal forms of FBF prompted the present study, whose aim was to generate new solid forms of this active pharmaceutical ingredient that might show potential for further development as medicinal candidates. Further motivation for this study was based on the recent finding that repurposing of fenbufen, in its role as an inhibitor of caspases, has been mooted in view of its ability to alleviate severe pancreatitis in a mouse model [31]. However, more research is required to support the safety of NSAIDs in this role [38].

A modest polymorphic screening exercise did not result in isolation of any new crystalline phases of FBF. However, the successful preparation and PXRD characterization of a γ-cyclodextrin inclusion complex were possible. In principle, this complex could improve FBF bioavailability and reduce adverse gastrointestinal effects caused by free FBF, but in vivo assessment is required to demonstrate this. Co-crystallization of FBF with numerous potential coformers resulted in isolation and full characterization of only a single co-crystal with isonicotinamide, which was found to have 1:1 stoichiometry, and despite many attempts to recover this crystal phase, it remained elusive, preventing assessment of its solubility (incidentally, based on the rule of thumb that the value of ΔpK_a_ = [pK_a_(base) − pK_a_ (acid)] can be used to predict whether a co-crystal or a salt will form [39], in the case of the acid fenbufen and the base isonicotinamide, the ΔpK_a_ value of −0.61 indicates that a co-crystal is more likely to form via their interaction).

Subsequent co-crystal screening with 19 coformers (including isonicotinamide) and three solvents, using both liquid-assisted grinding (LAG) and co-precipitation, resulted in only one LAG ‘hit’, with isonicotinamide as the coformer once again. With one exception, the co-precipitation experiments yielded FBF as the sole solid product, which is attributable to its very poor solubility in many solvents and consequent use of DMSO and/or pH adjustment to augment its solubility [40]. The exception was co-precipitation with MeCN as solvent, which yielded a crystal whose preliminary SCXRD analysis indicated a 2:3 FBF:isonicotinamide stoichiometry. The change in molar ratio from 1:1 to 1:1.5 resulted in the isolation of a pure, new product containing six molecular components. This unusual ionic co-crystal form was fully characterized and shown to enhance the aqueous solubility of FBF by a factor of ~300-fold, which is very significant. Furthermore, the solubility of FBF in the simulated intestinal fluid is ~10 times that of pure FBF in water. These favorable results indicate that the ionic co-crystal could be considered as a candidate for further formulation and development.

## Figures and Tables

**Table 1:** Onset temperature, peak temperature, and enthalpy values for endotherms in Figure 10.

Title 1	T_on_ (°C)	T_peak_ (°C)	ΔH (Jg^−1^)
FBF	186.0	187.0	159.1
ISONIC	155.8	157.4	209.2
IONIC CO-CRYSTAL	86.9	92.1	25.4
	135.3	136.4	121.5
	139.0	142.3	44.7
CO-CRYSTAL	128.5	143.3	118.8

**Table 2:** Solubilities of FBF and FBF content in the ionic co-crystal, in water and in FasSSIF.

Solid Phase	Water (mg/mL)	FaSSIF, pH 6.5 (mg/mL)
FBF	0.0022	6.92 ± 0.8
FBF in ionic crystal	0.68	8.65 ± 0.4

## Data Availability

Contact person M.R.C., mino.caira@uct.ac.za.

## Supplementary Materials

The following supporting information can be downloaded at https://www.mdpi.com/article/10.3390/pharmaceutics17070842/s1. CIF files for the two crystal structures were deposited at the Cambridge Crystallographic Data Centre: CCDC 2448175 (ionic co-crystal); CCDC 2448176 (co-crystal). Copies can be obtained free of charge on written application to CCDC, 12 Union Road, Cambridge, CB2 1EZ, UK (fax: +44-1223-336033), on request via e-mail to deposit@ccdc.cam.uk, or by access to http://www.ccdc.cam.ac.uk (accessed 1 May 2025). Table S1: Solvent media used in crystallization studies for polymorphic screening of FBF; Table S2: Fusion onset and peak temperatures and fusion enthalpies of solid products from polymorphic screening; Table S3: Description of methodology employed to complex FBF with native cyclodextrins; Figure S1. FT-IR spectra for different preparative treatments in attempts to complex FBF with γ-CD; Table S4: FT-IR spectral data indicating peak shifts resulting from different preparative treatments in attempts to complex FBF with γ-CD; Figure S2: DSC curves recorded following kneading of γ-CD and FBF in different molar ratios; Figure S3: Representative TGA and DSC curves for the fully hydrated γ-CD complex; Figure S4: HSM images of the FBF·ISONIC co-crystal at increasing temperatures Figure S5: TGA and DSC curves for the FBF·ISONIC co-crystal; Table S5: Crystal data and details of the structure determination for FBF·ISONIC co-crystal; Table S6: Outcomes of equimolar FBF + coformer LAG experiments with solvents EtOH, ethyl acetate and acetonitrile, based on PXRD analyses; Table S7: Crystal data and details of the structure determination of the FBIFISONIC ionic co-crystal; Figure S6: Composition of the ionic co-crystal and preparative method; Figure S7: HSM micrographs of significant events during heating of the ionic co-crystal at 2 K.min^−1^ while immersed in silicone oil; Figure S8: TGA and derivative curves for the ionic co-crystal; Figure S9: FT-IR spectra of the starting materials and the ionic co-crystal product.
