# Supplementary material for: Screening for Polymorphism, Cyclodextrin Complexation, and Co-Crystallization of the Non-Steroidal Anti-Inflammatory Drug Fenbufen: Isolation and Characterization of a Co-Crystal and an Ionic Co-Crystal of the API with a Common Coformer
Source: Pharmaceutics. 2025 Jun 27;17(7):842. doi: 10.3390/pharmaceutics17070842 (PMC12297964; doi:10.3390/pharmaceutics17070842)
Supplement: Supplementary file 1 [file pharmaceutics-17-00842-s001.zip › pharmaceutics-3656439-supplementary.pdf]

SUPPLEMENTARY MATERIAL

Screening for Polymorphism, Cyclodextrin Complexation and Co-crystallization of the Non-steroidal Anti-inflammatory Drug Fenbufen: Isolation and Characterization of a Co-crystal and an Ionic Co-crystal of the API with a Common Coformer

Hannah M. Frösler, Neo Refiloe Mancapa, Laura Catenacci, Milena Sorrenti, Maria Cristina Bonferoni, Mino R. Caira

---

Table S1: Solvent media used in crystallization studies for polymorphic screening of FBF.

	Solvent (ml/50 mg)	Yield (%)
MeOH	3	86
MeOH/H_2O 1:1 (v/v)	60	60
MeOH/H_2O 8:2 (v/v)	10	73
MeOH/H_2O 7:3 (v/v)	16	79
EtOH	5	51
EtOH/H_2O 1:1 (v/v)	30	74
EtOH/H_2O 8:2 (v/v)	3	77
EtOH/H_2O 7:3 (v/v)	7	84
Acetone	2	84
DMSO	1	51
Isopropanol	10	55
Acetonitrile	5	66
Ethyl acetate	5	80
Diethyl ether	40	74
Chloroform	7	72

NOTES: For each solvent, a sample of approximately 50 mg of commercial FBF was dissolved with heating to a temperature just below the respective boiling point to obtain a clear solution. The latter was left to recrystallize by spontaneous cooling at room temperature. The resulting solid crystals were filtered and dried in a dryer containing P_2O_5. The solid-state properties of the sample obtained were finally characterized.

Table S2: Fusion onset and peak temperatures and fusion enthalpies of solid products from polymorphic screening.

	T_{onset} (°C)	T_{peak} (°C)	ΔH_{melt} (J g^{-1})
FBF commercial	185.1 ± 0.6	185.4 ± 0.4	175 ± 1
MeOH	185.2 ± 0.3	185.4 ± 0.1	176 ± 1
MeOH/H_2O 1:1 (v/v)	185.2 ± 0.1	185.3 ± 0.1	175 ± 2
MeOH/H_2O 8:2 (v/v)	185.2 ± 0.1	185.2 ± 0.2	175 ± 2
MeOH/H_2O 7:3 (v/v)	185.3 ± 0.5	185.6 ± 0.6	173 ± 1
EtOH	185.2 ± 0.3	185.4 ± 0.5	176 ± 3
EtOH/H_2O 1:1 (v/v)	185.2 ± 0.1	185.3 ± 0.2	176 ± 1
EtOH/H_2O 8:2 (v/v)	185.2 ± 0.2	185.6 ± 0.1	176 ± 2
EtOH/H_2O 7:3 (v/v)	185.3 ± 0.1	185.3 ± 0.1	176 ± 4
Acetone	185.1 ± 0.1	185.4 ± 0.3	173 ± 1
DMSO	184.7 ± 0.4	185.3 ± 0.3	168 ± 3
Isopropanol	185.0 ± 0.1	185.2 ± 0.1	171 ± 1
Acetonitrile	185.4 ± 0.6	185.6 ± 0.1	175 ± 1
Ethyl acetate	185.5 ± 0.1	185.7 ± 0.6	172 ± 2
Diethylether	186.1 ± 0.5	186.6 ± 0.4	180 ± 2
Chloroform	185.2 ± 0.5	185.6 ± 0.1	173 ± 1

TABLE S3: Description of methods employed to complex FBF with native cyclodextrins

**PM** -> Physical mixtures were prepared by homogeneously mixing equimolar amounts of FBF with each native CD, previously sieved to collect the particle size fraction < 250 µm, in a turbula mixer for 20 min.
**KN** -> Each PM was wetted in a mortar with a few drops of an ethanol:water solution (1:1 v/v) or of a water solution and then dried at 70 °C to a constant mass in an oven. This procedure was repeated three times and the samples were then sieved through a 250 µm sieve.
**CP** -> FBF was dissolved in ethanol and the resultant solution was added dropwise to an equimolar amount of CD previously dissolved in an aqueous solution, under magnetic stirring for 2 hours at room temperature or by heating at 80 °C. The resulting solution or suspension was maintained at room temperature for 24 hours or 7 days and the resulting solid phase was filtered and subsequently dried up to constant weight.
**RV** -> PTB was dissolved in 6 ml of ethanol and added dropwise to 2 ml aqueous solution of each CD. The suspension was stirred at 80 °C until to obtain a clear solution and subsequently evaporated using a rotary<br>evaporator under reduced pressure (RV).

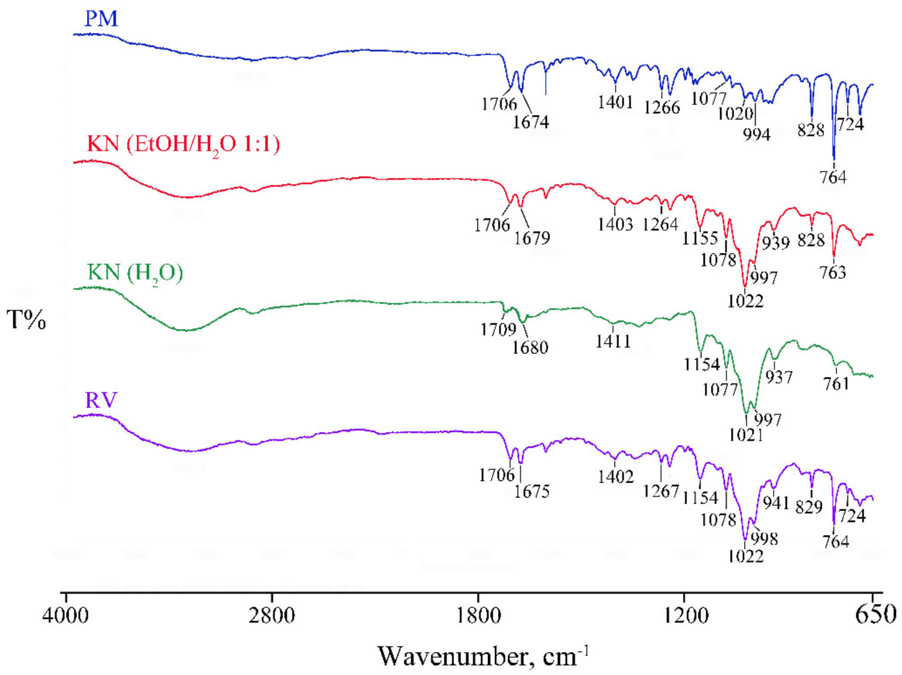

Figure S1. FT-IR spectra for different preparative treatments in attempts to complex FBF with γ-CD.

Table S4: FT-IR spectral data indicating peak shifts resulting from different preparative treatments in attempts to complex FBF with γ-CD.

	FBF	γ-CD	PM	KN EtOH/ H_2O 1:1 (v/v)	KN H_2O	RV
Wavenumber (cm^{-1})	1705		1706	1706	1709	1706
	1674		1674	1679	1680	1675
	1401		1401	1403	1411	1402
	1266		1266	1264	X	1267
		1151	X	1155	1154	1154
		1077	1077	1078	1077	1078
		1021	1020	1022	1021	1022
		997	994	997	997	998
		944	X	939	937	941
	828		828	828	X	829
	764		764	763	761	764
	724		724	X	X	724

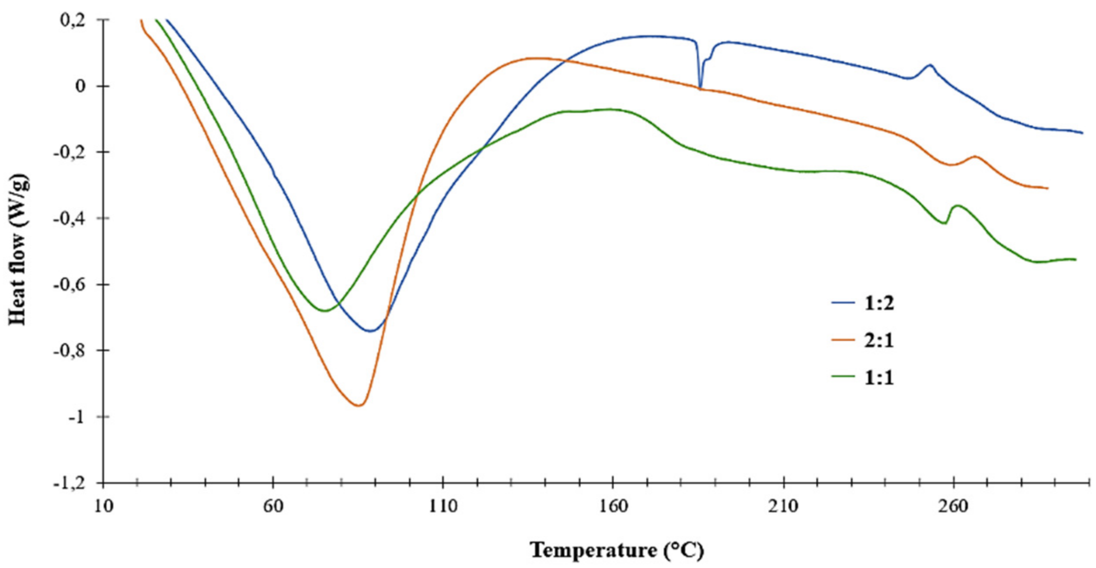

Figure S2: DSC curves recorded following kneading of γ-CD and FBF in different molar ratios.

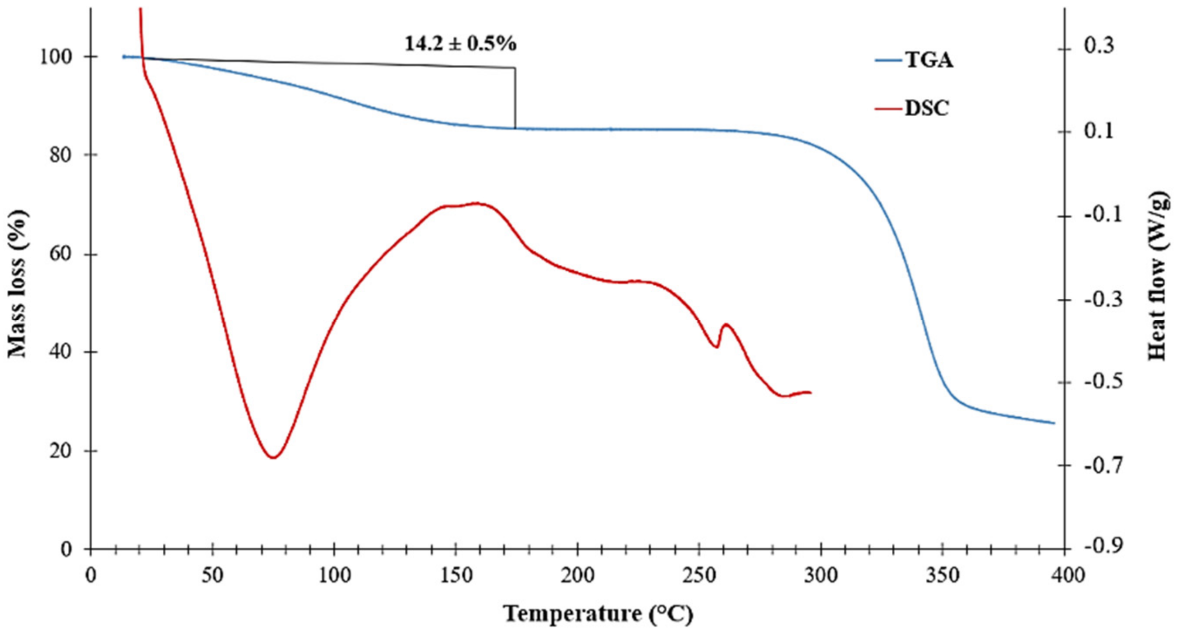

Figure S3: Representative TGA and DSC curves for the fully hydrated γ-CD complex.

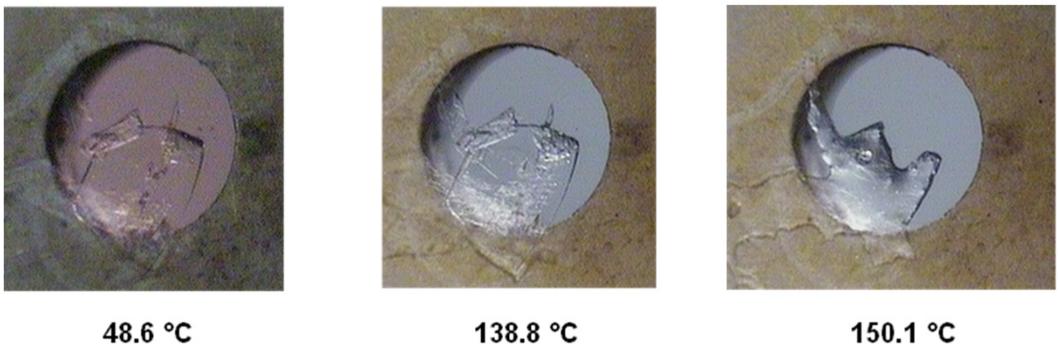

Figure S4: HSM images of the FBF·ISONIC co-crystal at increasing temperatures.

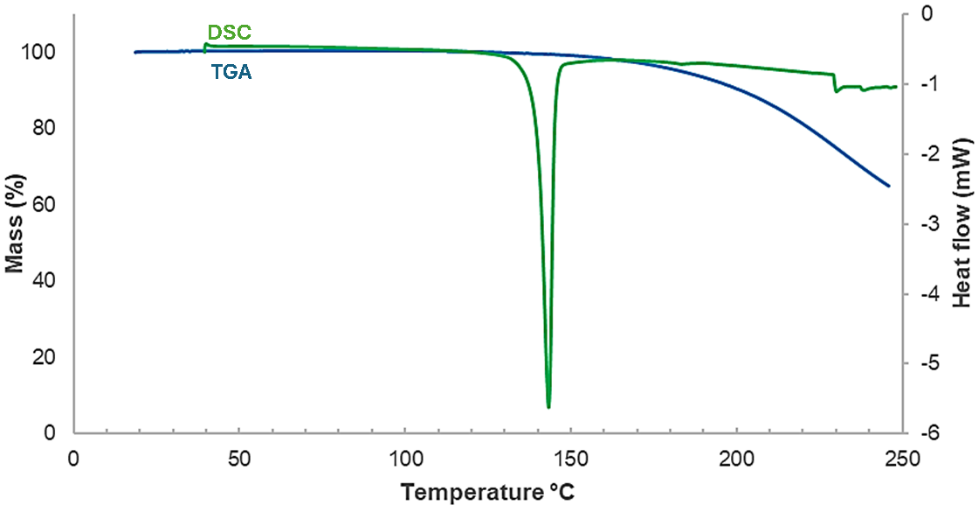

Figure S5: TGA and DSC curves for the FBF·ISONIC co-crystal.

TABLE S5: Crystal Data and Details of the Structure Determination for FBF·ISONIC co-crystal P(-1), R = 0.05

Crystal Data	
Formula	$C_{16}H_{14}O_3$, $C_6H_6N_2O$
Formula weight	376.40
Crystal system	triclinic
Space group	P-1 (No.2)
a, b, c (Å)	5.9181(1) 10.8813(17) 29.701(5)
α, β, γ (°)	83.638(4) 86.223(4) 80.617(4)
V (Å^3)	1873.2(5)
Z	4
D_{calc} (g/cm^3)	1.335
μ(MoKα) (mm^{-1})	0.093
F(000)	792
Crystal size	0.07 x 0.15 x 0.60
**Data-collection**	
Temperature (K)	173
Radiation (Å)	MoKα, 0.71073
θ-min, θ-max (°)	1.4, 27.5
Dataset	-7: 7; -14: 14; -38: 38
Tot., Uniq., Data, R(int)	48576, 8611, 0.052
Observed Data [I > 2.0σ(I)]	5668
**Refinement**	
N_{ref}, N_{par}	8611, 515
R, wR2, S	0.0459, 0.1278, 1.03
Weighting scheme	w=1/[\s^2^(Fo^2^)+(0.0556P)^2^+0.2979P] where P=(Fo^2^+2Fc^2^)/3
Max. and Av. Shift/Error	0.00, 0.00
Min. and Max,. Resd. Dens. (eÅ^{-3})	-0.22, 0.25

TABLE S6: Outcomes of equimolar FBF + coformer LAG experiments with solvents EtOH, ethyl acetate and acetonitrile, based on PXRD analyses.

COFORMER	PRODUCT
Adipic acid	Physical mixture
Benzamide	Physical mixture
Citric acid	Physical mixture
Ferulic acid	Physical mixture
Fumaric acid	Physical mixture
Glycolamide	Physical mixture
Hippuric acid	Physical mixture
4-hydroxybenzoic acid	Physical mixture
Isonicotinamide	New phase
Lactamide	Physical mixture
L-Malic acid	Physical mixture
Orotic acid	Physical mixture
Oxalic acid	Physical mixture
Pimelic acid	Physical mixture
Pyrazinecarboxamide	Physical mixture
Saccharin	Physical mixture
Suberic acid	Physical mixture
Succinic acid	Physical mixture

TABLE S7: Crystal Data and Details of the Structure Determination for the FBFISONIC ionic co-crystal $P2_1/c$, R = 0.0

Crystal Data	
Formula	$C_{16}H_{14}O_3$, $C_{16}H_{13}O_3^{1-}$, $2(C_6H_6N_2O)$, $C_6H_7N_2O^{1+}$, H_2O
Formula weight	892.94
Crystal system	monoclinic
Space group	$P2_1/c$ (No. 14)
a, b, c (Å)	11.4620(17) 7.5967(12) 48.860(7)
α, β, γ (°)	90 90.096(4) 90
V ($Å^3$)	4254.4(11)
Z	4
D_{calc} (g/cm^3)	1.394
μ(MoKα) (mm^{-1})	0.098
F(000)	1880
Crystal size	0.08 x 0.13 x 0.26
**Data-collection**	
Temperature (K)	100
Radiation (Å)	MoKα, 0.71073
θ-min, θ-max (°)	2.0, 28.3
Dataset	-14: 15; -10: 10; -65: 60
Tot., Uniq., Data, R(int)	68268, 10232, 0.030
Observed Data [I > 2.0σ(I)]	9345
**Refinement**	
N_{ref}, N_{par}	10232, 602
R, wR2, S	0.0626, 0.1381, 1.06
Weighting scheme	w=1/[\s^2^(Fo^2^)+(0.0360P)^2^+6.6582P] where P=(Fo^2^+2Fc^2^)/3
Max. and Av. Shift/Error	0.00, 0.00
Min. and Max,. Resd. Dens. ($eÅ^{-3}$)	-1.03, 0.77

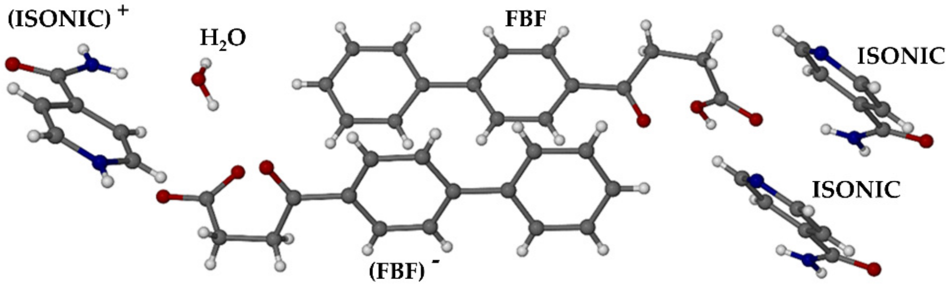

SYNTHESIS: Single crystals of the ionic co-crystal were synthesised using FBF (5.0 mg, 0.012 mmol) and ISONIC (3.6 mg, 0.018 mmol) each dissolved in 0.6 ml of acetonitrile. The FBF solution was added to that of ISONIC, stirred at 55 ± 5 °C for 6 hours and filtered thereafter. For product crystallization, the slow cooling method was followed: the filtered solution was sealed and placed in a Dewar flask containing water at 55 °C. After 3 days, the insulation was removed, and single crystals were isolated for X-ray data-collection.

Figure S6: Composition of the ionic co-crystal and preparative method.

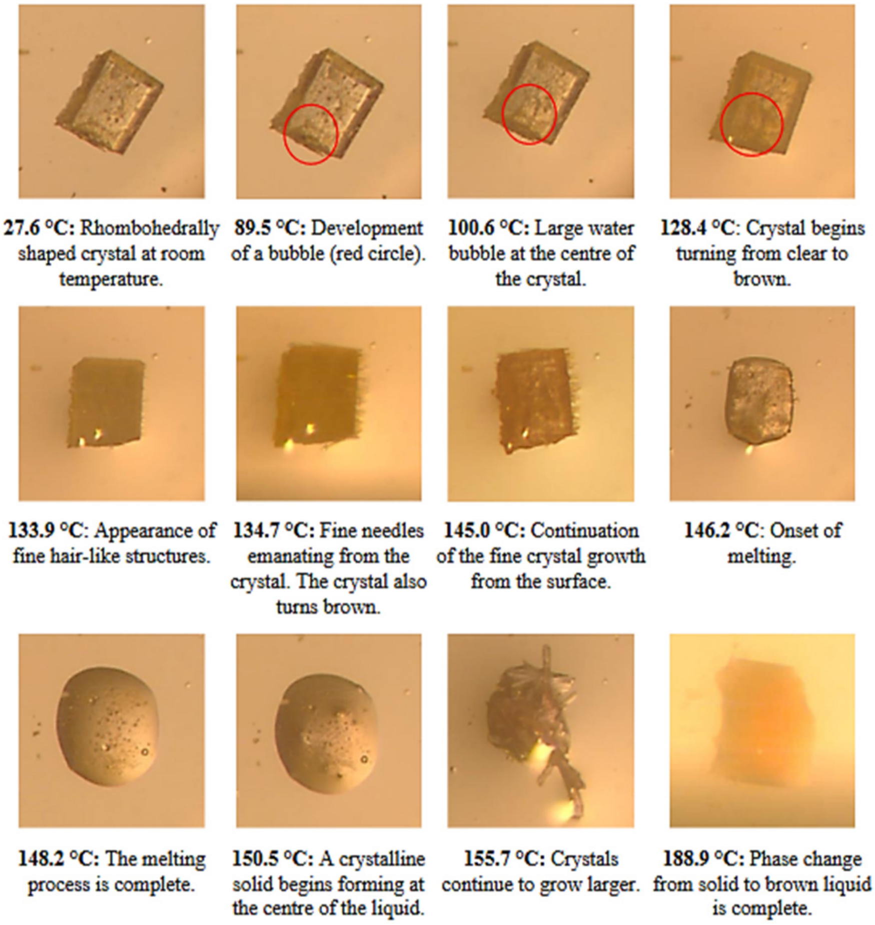

Figure S7: HSM micrographs of significant events during heating of the ionic co-crystal at 2 $K.min^{-1}$ while immersed in silicone oil.

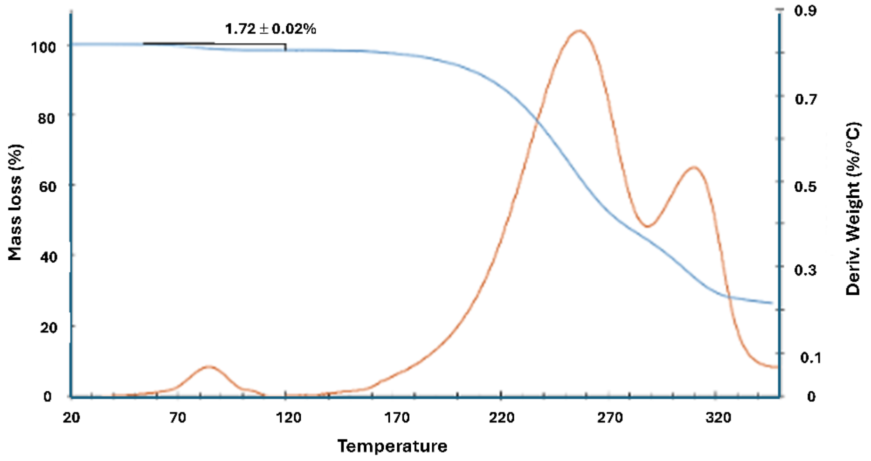

Figure S8: TGA and derivative curves for the ionic co-crystal.

NOTES: Significant events include dehydration (onset at ~58 °C), a mass loss of 52.5 ± 0.5% from 152 °C, and a final decomposition with onset temperature ~280 °C with a 19.7 ± 0.3% mass loss.

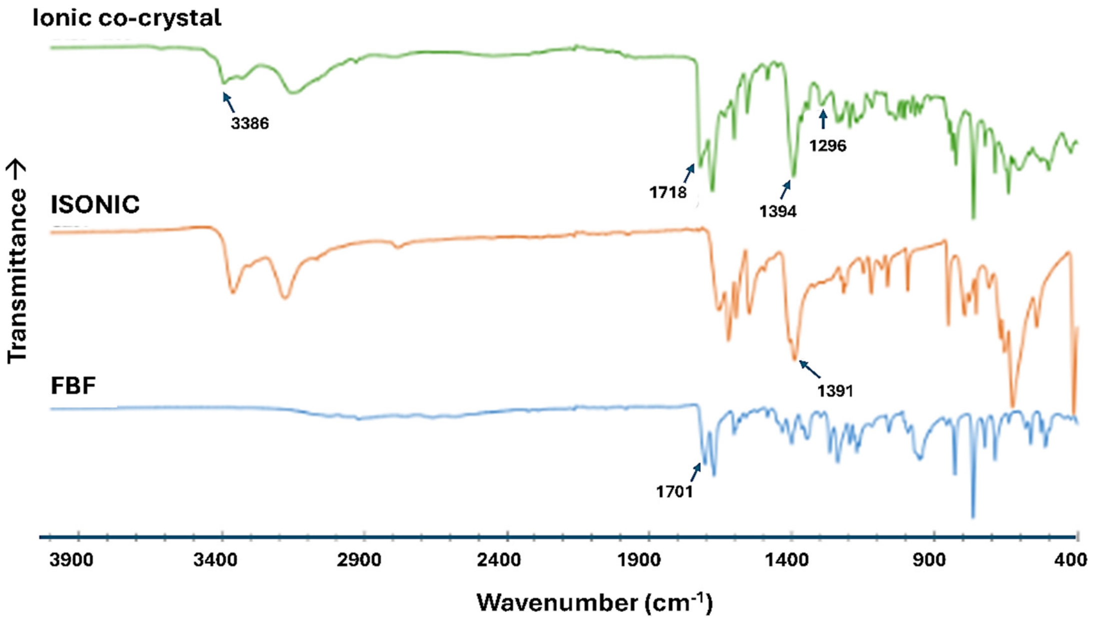

Figure S9: FT-IR spectra of the starting materials and the ionic co-crystal product.

NOTES: Band at 1701 cm^{-1} for carboxyl C=O in FBF shifted to 1718 cm^{-1} in the product. Small band shift from 1391 to 1394 cm^{-1} assigned to O-H bending; weak band at 1296 cm^{-1} in the salt product indicates C-O stretching in the product; asymmetric and symmetric stretching bands at 3350 and 3190 cm^{-1} in pure ISONIC shifted to higher frequencies in the product.
